# Severe renal Fanconi and management strategies in Arthrogryposis-Renal dysfunction-Cholestasis syndrome: a case report

**DOI:** 10.1186/s12882-018-0926-1

**Published:** 2018-06-15

**Authors:** Alejandra Rosales, Maissa Mhibik, Paul Gissen, Oscar Segarra, Susana Redecillas, Gema Ariceta

**Affiliations:** 1 Pediatric Nephrology, Hospital Universitario Vall d’Hebron, Passeig de la Vall d’Hebron, 119-129, 08035 Barcelona, Spain; 2 Department of Pediatrics 1, Medical University of Innsbruck, Innsbruck, Austria; 3 MRC Laboratory for Molecular Cell Biology, University College London, London, UK; 4 Pediatric Gastroenterology, Hospital Universitario Vall d’Hebron, Barcelona, Spain

**Keywords:** Arthrogryposis-Renal failure-Cholestasis syndrome (ARC), VPS33B, VIPAR, Proximal tubular acidosis, Renal Fanconi, Ichthyosis, Cholestasis, Pruritus, LDL-apheresis

## Abstract

**Background:**

Arthrogryposis-Renal dysfunction-Cholestasis syndrome (ARC, MIM#208085) is a rare multisystem disease due to mutations in the *VPS33B* and *VIPAR* genes, both involved in maintaining apical-basolateral cell polarity. The correlation between mutations and phenotype in the ARC Syndrome is not well described. We report on a 6 year old patient who presented with severe renal Fanconi as first manifestation of ARC related to a combined de novo mutation in the *VPS33B* gene.

**Case presentation:**

A 6 year old girl presented during the first year of life with severe renal Fanconi as the first manifestation of ARC-Syndrome. This case presents all defining features of ARC syndrome (including liver, skin and articular manifestations) with predominantly renal impairment at presentation. This novel mutation may be associated with a pronounced renal phenotype in ARC. Furthermore, we report on the successful use of LDL-Apheresis and biliodigestive derivation for treatment of cholestatic pruritus with encouraging results.

**Conclusion:**

ARC is a heterogeneous disorder with early mortality. This case report contributes to a better understanding of this rare disorder, describes a novel mutation in the VPS33B gene and presents an innovative rescue treatment approach.

## Background

Arthrogryposis-Renal dysfunction-Cholestasis syndrome (ARC, MIM#208085) is a rare multisystem disease due to mutations in the *VPS33B* and *VIPAR* genes, both involved in maintaining apical-basolateral cell polarity. *VPS33B* is involved at multiple stages of regulation of vesicular membrane fusion and intracellular trafficking. Cell polarity is crucial for the function of proximal tubular cells (PTC) and altered distribution of apical proteins could lead to proximal tubule dysfunction and renal Fanconi syndrome (RFS) in ARC patients [1]. We report on a (now 6 year old) patient who presented with complete RFS as first manifestation of ARC related to a combined de novo mutation in the *VPS33B* gene.

## Case report

The patient manifested at 2 months of age with fever and irritability. The girl, born at term, was initially clinically unremarkable apart from bilateral vertical talus. A normal karyotype was found at prenatal testing. The parents, who were not consanguineous, reported a positive family history for a de novo mutation in the *PAX 2* gene (cousin).

At the age of 2 months, initial investigations showed normal blood count and biochemistry (serum glucose 90 mg/dL, Urea 13 mg/dL, Creatinine (Cr) 0.3 mg/dL, normal electrolytes -Na 139.3 mmol/L, K 4.8 mmol/L, Cl 107 mmol/L-, Calcium 10.5 mg/dL, P 4.7 mg/dL), apart from reduced serum uric acid (1 mg/dL) and increased alkaline phosphatase (1567 U I/L). In addition, urine dipstick demonstrated glycosuria and proteinuria. Low urine osmolarity (UOsm 175 mOsm/kg) and estimated glomerular filtration rate (eGFR) of 30.8 mL/min/1.73m^2^ were observed. Uranalysis showed RFS with glycosuria (Uglucose/Cr 13.6 mg/mg), hyperphosphaturia (19.9 mg/kg/day), low phosphate reabsorption (TRP 77.46%, TmP/GFR 3.32 mg/dL), hypercalciuria (UCa/Cr 0.57 mg/mg; VCa 7 mg/kg/day) with low molecular weight proteinuria and global aminoaciduria (Uprotein/Cr 7.7 mg/mg; V protein 36.1 mg/m2/h, β2microgb 30 mg/m2/h, MAU/Cr ratio 0.6 mg/g) in the absence of sodium wasting (C.Na 0.44%) or metabolic acidosis (pH 7.40, pCO_2_ 45.2 mmHg, Bicarbonate 27.4 mmol/L). Cystinosis, the most common cause of RFS, could be rapidly ruled out after establishing the presence of a normal leukocyte cystine level. Other causes of inherited RFS were excluded. Renal ultrasound (US) demonstrated moderately hyperechogenic normal sized but abnormally structured kidneys (Patient clinical characteristics are described in Table 1).

**Table 1:** Summary of patient clinical characteristics

Renal involvement	
Renal Fanconi	
*Presentation: Serum Biochemistry: glucose 90 mg/dL, Urea 13 mg/dL, Creatinine (Cr) 0.3 mg/dL, normal electrolytes -Na 139.3 mmol/L, K 4.8 mmol/L, Cl 107 mmol/L-, Calcium 10.5 mg/dL, P 4.7 mg/dL, eGFR 30.8 ml/min/1.73m* ^*2*^ *, diuresis 2.6 ml/kg/h, urine osmolarity 175 mOsm/kg, glycosuria (Uglucose/Cr 13.6 mg/mg) hyperphosphaturia 19.9 mg/kg/day, TRP 77.46%, TmP/GFR3.32 mg/dL, hypercalciuria 7 mg/kg/d, UCalcium/creatinine ratio 0.57 mg/mg proteinuria Uprot/Cr 7.7; β2microgb 30 mg/m2/h, MAU/Cr ratio 0.6 mg/g, aminoaciduria C.Na 0.44% Absence of metabolic acidosis*	
Recurrent episodes of dehydration and decompensation of renal Fanconi	
Carnitine deficiency	
Serum Cu within normal limits	
Secondary Hyperparathyroidism	
Small kidneys, abnormal structure, absence of nephrocalcinosis since 2 years of life	
Progressive CKD from 2 years of life	
Skeletal features	
Bilateral vertical talus	
Hip dysplasia	
Arthrogryposis	
Bone age retardation	
Impaired growth, partial response to rHGH	
Neurological involvement	
Bilateral hypoacusia	
Developmental delay	
Corpus callosum agenesia/hypoplasia	
Gastrointestinal involvement	
Feeding difficulties (tube feeding and button gastrostomy)	
Gastro-Esophageal Reflux	
Cow’s Milk intolerance	
Recurrent Transaminitis	
Cholestasis (increased serum bile acids with normal bilirubin)	
Skin involvement	
Skin lesions (Lamellar Ichthyosis), pigmentation	
Pruritus	
Hematology/Infection	
Iron-deficiency Anemia	
Recurrent Sepsis caused by *Staphylococcus aureus*	
Bleeding episodes, platelet dysfunction (prolonged bleeding time, reduced aggregability)	

Over the first year of life, the patient suffered from increasing severity of RFS with overt polyuria (6 mL/kg/h), proteinuria, hypercalciuria and stagnation in weight gain. It came to recurrent severe episodes of diarrhoea with vomiting, dehydration and considerable electrolyte derangements: high fluid losses made management challenging, requiring frequent hospitalization. In order to fulfil nutritional requirements, and to replace fluid- and electrolyte losses, tube feeding and finally gastrostomy became necessary. Added to these problems was the impaired ability to concentrate urine, as previously mentioned. At this stage the presence of overt RFS, female gender, and progressive CKD, was regarded as sufficient explaination for the overall clinical picture, and primary nephrogenic diabetes genetic testing was not yet considered.

Treatment with indomethacin, hydrochlorothiazide at low doses, and later on, at 4 years of age, desmopressin, led to a decrease in urine output, small size hyperechogenic kidneys and CKD III became evident, even in the absence of nephrocalcinosis.

Furthermore, the patient presented with a distinctive phenotype with slight hypertelorism, episodic mild skin tanning (without jaundice) and bone anomalies (vertical talus, hip dysplasia), short stature with partial response to treatment with rHGH. Otoacoustic emissions showed bilateral hypoacusia, a cerebral MRI displayed corpus callosum hypoplasia. Blood smear did not show remarkable findings. However, platelet dysfunction (with normal coagulation tests and platelet account, but prolonged bleeding time, and reduced platelet aggregability) was also detected. Bleeding episodes were observed in the course of minor procedures and could be successfully managed with platelet transfusions. Platelets were not studied by electron microscopy.

**Fig. 1:**
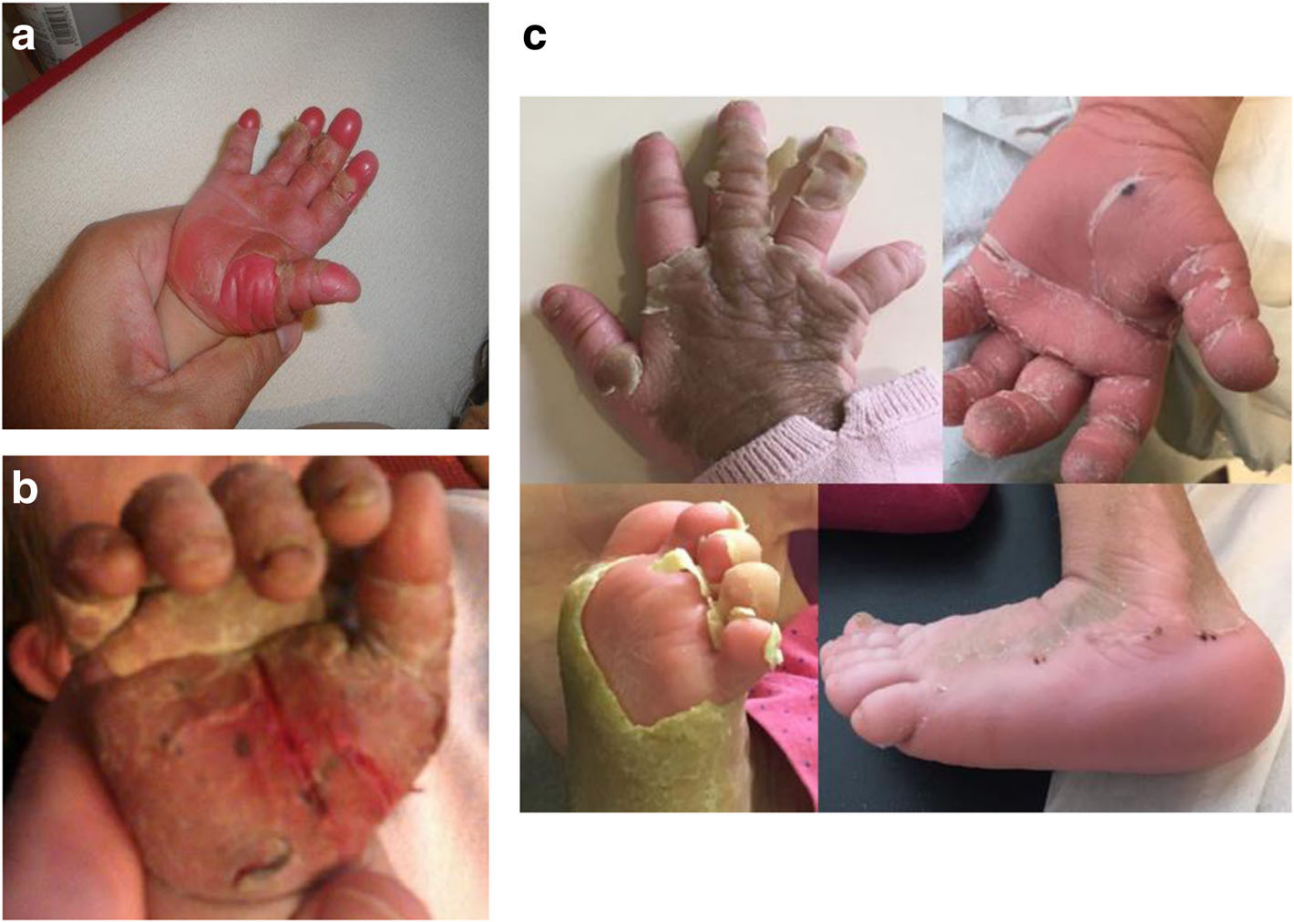
Skin manifestations. **a** at presentation. **b** before biliodigestive anastomosis. **c** after biliodigestive anastomosis

At the end of the second year of life, progressively, the patient had developed refractory pruritus that prevented her from sleeping and provoked severe anxiety, explaining in part the developmental delay, and bleeding episodes due continuous scratching. In addition, recurrent skin infections and several bacteraemia episodes due to *Staphylococcus aureus* occurred and were successfully treated with parental antibiotics. Over time, she developed increasing cutaneous changes in terms of ichthyosis, characteristically mainly involving feet and hands, nails and with time plantar and palmar oedema (Fig. 1). Skin biopsy showed pronounced hyperkeratosis.

In addition to remarkable skin manifestations, during those first 2 years of life, the patient presented with an intermittent transaminitis not attributable to indomethacin (ASAT max~1000UI/L, ALAT max~800UI/L, Gamma-GT normal), without jaundice but persistently normal serum bilirubin levels. Global liver function and ultrasound remained normal. However, remarkable elevation of total bile acids (tBA) (> 100 μmol/L) in plasma compatible with severe cholestasis was detected, which explained patient’s severe pruritus and partial response to bile salts binders as Cholestyramine. In summary, our patient presented with all of the characteristic signs and symptoms of ARC (summarized in Table 1).

The clinical suspicion of ARC Syndrome was confirmed through genetic analysis which showed two de novo mutations in the *VPS33B* gene: an heterozygous mutation (intron 16–17) c.1225 + 5G > Cp.(?) (associated to moderate phenotype) [1], and second novel heterozygous mutation (exon 7) c.440_499del p.(Pro147Argfs*4) (premature stop codon, severe mutation, not yet described) (analysis performed at the MRC Laboratory for Molecular Cell Biology, University College London, UK, by manuscript authors Maissa Mhibik and Paul Gissen).

The patient is now 6 years old, with impaired renal function - eGFR of ~ 30 mL/min/1, 73 m2 - and severe RFS with polyuria requiring high amounts of fluids and electrolytes. Skin lesions and pruritus remain one of the leading symptoms with persistent transaminitis and normal liver synthesis function. Various treatment approaches (including barbiturates and antipsychotics) led to no success, but administration of high doses of ursodeoxycholic acid could achieve a partial response, supporting the pathogenic role of cholestasis in dermatologic abnormalities. As a proof of concept that removal of bile acids could improve severe pruritus and skin manifestations [2], at the age of 5 years, the child started treatment with LDL-apheresis using an immunoadsorption based method (Life18TM, LDL-Pro Columns, Miltenyi Biotec). The procedure was performed twice weekly for a total of 24 sessions, using regional anticoagulation with citrate. Each session lasted 1.5 h and a volume equivalent to 2 patient-volemia was treated. The treatment was perfectly tolerated and no complications, especially in terms of bleeding, were observed. A significant decrease in tBA levels before and after treatment with LDL-apheresis was achieved (*p* < 0.001 (t test), Table 2), with median pre-treatment tBA of 41 μmol/L (IQR 19.5) and post-treatment tBA of 20.7 μmol/L (IQR 8.4). Reduction of the pruritus could be achieved, and skin lesions improved partially too, under pharmacological treatment with ursodesoxycholic acid. Driven by that result, in January 2017 an entero-biliary anastomosis was performed. Shortly after the intervention we observed a significant improvement of pruritus, regression of skin lesions, and significant improvement in quality of life (Fig. 1).

**Table 2):** Total bile acids (tBA) levels under medical treatment, during LDL-apheresis and after biliodigestive anastomosis. (median, IQ)

	Medical treatment	LDL-Apheresis		Biliodigestive anastomosis
		Pre	Post	
tBA^a^	87.3 (48–117)	41 (32.5–52)	20.7 (17–25.4)	23.6 (22.6–28)
Colesterol	102 (99–132.6)	148 (142–159)	61 (59–68.5)	126 (118.5–143)
LDL	77 (73.5–80.5)	77 (73–78)	16 (15.5–17.5)	68 (58.5–77.5)
HDL	33 (32–34.5)	44 (43–49)	28 (27.5–33.5)	26.5 (23.75–27.25)

^a^Reference values: tBA < 10 μmol/L

## Discussion

Polarization of human epithelial cells is crucial for adequate organ development and function. The PT is the site of reabsorption of valuable solutes. Disruption of endocytosis and intracellular trafficking can lead to impaired PT function and present as RFS. The *VPS33B* gene is involved at multiple stages in the regulation of vesicular membrane fusion and trafficking, including maturation and control of endosomes [1, 3]. In the PT, disturbed endosome maturation could affect the apical recycling pathway [4]. Most ARC patients present certain grade of tubular dysfunction with proteinuria, aminoaciduria, glycosuria and tubular acidosis which can worsen with intercurrent infectious events [1]. The correlation between genotype and phenotype in ARC is not well described. We present a 6-year old patient with RFS as first manifestation of ARC and two different de novo mutations in the *VPS33B* gene. The *VPS33B* c.1225 + 5G-C mutation seems to affect the ability of the complexes to colocalize properly on tubular-vesicular recycling membranes, which would impair, at least partially, their cellular functioning [3, 4]. No reports describing the second and novel identified mutation have been found so far ((exon 7) c.440_499 del p. (Pro147Argfs*4)). This mutation may be severe since it introduces a premature stop codon and could be related to a pronounced renal phenotype of ARC syndrome.

Most reported ARC cases describe severe forms and early mortality [5]. Gissen et al. report on 62 ARC patients of 35 families, none of the patients was older than 20 months at the time of publication. However, it has been described that affected individuals with the *VPS33B* c.1225 + 5G-C mutation, may present a somehow milder phenotype [1]. Despite disease severity, and multidisciplinary management, our patient survival beyond 6 years of age could be partially attributed to that. Besides severe infections, bleeding is one of the most relevant factors affecting mortality in ARC patients [3], and indeed constituted one major morbidity factor in our patient before her specific diagnosis was identified. *VPS33B* is involved in the formation of precursor alpha-granules of platelets (both stored and membrane components) and presentation is similar to gray platelet syndrome [6]. Currently we are avoiding bleeding complications using preventive platelet transfusions before invasive procedures in our patient, or as early treatment of haemorrhage episodes, with good outcome.

ARC patients present a variety of skin and musculoskeletal alterations and seem to share a peculiar phenotype. *VPS33B* plays a role in collagen maturation (important component of the basal membrane, also crucial in the development of cell polarity) and keratinocyte differentiation (metabolism of lamellar bodies) [7, 8]. Furthermore, disturbed hepatocyte polarity and elevation of biliary acids are involved in the development of skin lesions and pruritus. Recently, we published the beneficial results of LDL-apheresis in this patient, as an example of potential indication of the procedure in patients with refractory cholestatic pruritus [9]. This case showed drastic improvement of skin lesions after biliodigestive anastomosis (Fig. 1). Similar results were already reported after liver transplantation in a 12 year old ARC patient [10]. Nevertheless, a previous report shows response of pruritus to cutaneous biliary diversion but persistence of severe skin lesions [3].

## Conclusion

ARC is a heterogeneous disorder with early mortality. This case report contributes to a better understanding of this rare disorder, describes a novel mutation in the *VPS33B* gene which could be associated to a pronounced renal phenotype in ARC-Syndrome. Furthermore, we present LDL-apheresis as an innovative rescue treatment approach for cholestasis associated pruritus in ARC-Syndrome.

## Abbreviations

ARC: Arthrogryposis-Renal-Cholestasis Syndrome
eGFR: Estimated glomerular filtration rate
PTC: Proximal tubule cells
RFS: Renal Fanconi Syndrome
rHGH: Recombinant human growth hormone
US: Ultrasound
